# Hemogram and iron indices in renal anemia and the amelioration with *Carica papaya* leaf extract applied on albino rat model

**DOI:** 10.1042/BSR20181699

**Published:** 2019-04-26

**Authors:** Ibtsam Gheith, Abubakr El-Mahmoudy

**Affiliations:** 1 Department of Clinical Laboratory Sciences, Faculty of Applied Medical Sciences, Taibah University, 344 Medinah, K.S.A.; 2 Department of Biotechnology, Animal Health Research Institute, 11843 Dokki, Egypt; 3 Department of Pharmacology, Benha University Faculty of Vet. Medicine, 13736 Moshtohor, Qalioubeya, Egypt

**Keywords:** blood homeostatic, Carica papaya, leaves, nephroprotective, renal disorders

## Abstract

The present study was designed to look at the hematological disorders in gentamicin nephrotoxicity model, as kidney is considered as one of the hemopoietic organs. In a previous study, novel and classical kidney injury biomarkers were utilized to evaluate the nephroprotective potential of *Carica papaya* leaf extract (*CPLE*) in the same model in albino rats. Gentamicin (100 mg/kg, subcutaneously, for 21 consecutive days) resulted in significant decreases in red blood cell (RBC) count, hemoglobin concentration (HGB), and packed cell volume (PCV) value, with minimal alterations in erythrocytic indices. Leucogram showed leukocytosis, granulocytosis, and thrombocytopenia. Erythropoietin (EPO) levels were also drastically decreased by the end of the experimental course. Serum iron, unsaturated iron-binding capacity (UIBC), total iron binding capacity (TIBC), transferrin saturation %, and serum transferrin concentration values were significantly decreased in contrast to ferritin, which was increased. When concurrently administered with gentamicin, *CPLE* (150 and 300 mg/kg, orally via gastric tube, for 21 days) significantly protected against the drastic effects of the former on the blood profile with improving potentials on erythrogram, leukogram, thrombocytes, EPO, iron and its indices, in a dose-dependent manner. These data may suggest *CPLE* as an appreciated blood homeostatic and nephroprotective agent from a natural source that could be a good remedy in conditions associated with blood disorders.

## Introduction

In addition to its major multiple functions regarding clearance and electrolyte and acid–base balances, kidney plays a major role in the process of hematopoiesis as it is responsible for erythropoietin (EPO) synthesis and release. EPO is produced by some cells located in the renal cortex that is the main site getting damaged by repeated administration of aminoglycosides, including gentamicin [1].

EPO is a glycoprotein hormone that is the initiator and regulator of erythropoiesis. It is produced primarily by renal peritubular cells related to proximal convoluted tubules [2,3]. Anemia associated with kidney injury, inflammation, and failure is attributed to inadequate EPO production and the shortened erythrocyte life span due to uremic toxins that are accumulated in the blood because of renal dysfunction [4].

In addition to EPO, iron deficiency may contribute to development of anemia in inflammatory kidney disease [5]. Iron deficiency may be absolute or functional; the former is uncommon in kidney disease (it only exists when supplementation of iron is deficient in diet) and is diagnosed when serum iron as well as ferritin (protein that stores iron) and transferrin (the plasma iron carrier that is the only source of iron for erythropoiesis) saturation % are all lower than the physiological levels. However, functional iron deficiency indicates restriction of the availability of iron for erythropoiesis despite the normal or higher (most common) levels of iron stores (ferritin) in the body. Hepcidin (a peptide hormone synthesized in hepatocytes that is a key regulator of iron levels) decreases the efflux of recycled iron from splenic and hepatic macrophages, and the release of iron from storage in hepatocytes leading to functional iron deficiency. Moreover, it binds to enterocytes causing poor absorption of dietary iron. Inflammation (including kidney injury) increases hepcidin concentration, along with other inflammatory mediators as C-reactive protein and Interleukin-6, which results in lower levels of circulating iron and development of anemia [6].

As kidney is the main organ for eliminating xenobiotics that may adversely affect its function; therefore, continuous trials are adopted to find out safer medicaments with minimal or no nephrotic side effects yet be effective in improving renal function. In this respect, pharmacologists search effectively in the scope of medicinal natural products with good impacts on urinary system. Among those is *Carica papaya*, which is a medicinal plant that is distributed in tropical areas and is being used for its nutraceutical and pharmacological potentials in traditional medicine [7].

Kidney disease and its severity are diagnosed in the laboratory by determination of renal biomarkers, including the standard conventional markers (urea and creatinine) and novel ones (e.g., kidney injury molecule, clusterin, etc.). In a previous study [8], we have evaluated the nephroprotective role of aqueous extract *Carica papaya* in gentamicin-induced nephrotoxicity based on both classical and novel biomarkers. In the present study, we are trying to assess the possible modulating role of this plant extract on blood parameters (erythrogram and leukogram) and to clarify the underlying mechanisms of such modulation, if any, by determination of serum EPO, iron, unsaturated iron-binding capacity/total iron-binding capacity (UIBC/TIBC), transferrin, transferrin saturation %, and ferritin in kidney disease using the same model with some modifications.

## Materials and methods

### Plant part used and extraction procedure

**Figure 1:**
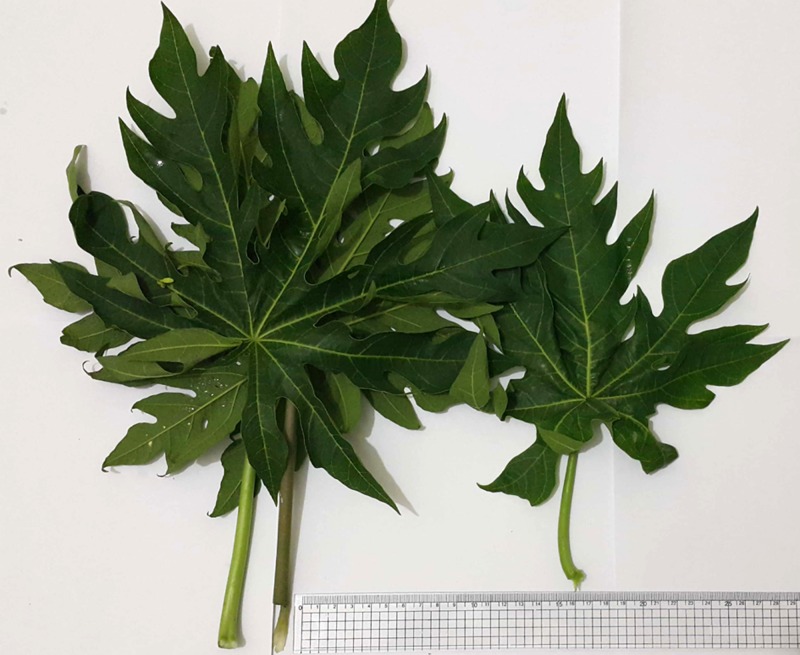
*Carica papaya* leaves used for extract preparation

As described in a previous study [8], the fresh leaves of *Carica papaya* (Figure 1) were used after collection from our local environment and identification by a Botany specialist. The adopted methodological procedures of extraction were modified later [9]. Plant leaves were refluxed in running tap water and then with bi-distilled water, shade dried at room temperature, and chopped using clean knife and flat board. The extract was prepared by macerating a weighed amount (200 g) of the small parts of the leaves in a known volume (1.5 l) of aqueous: organic solvent (bi-distilled water: ethanol, 70:30, *v/v*) in covered Erlenmeyer flasks. Maceration continued for 48 h under refrigeration with occasional shaking. The hydroethanolic extract was filtered and then concentrated using a gently shaking water bath at 56°C in clean, pre-weighed, labeled glass beakers. The weight of the obtained semisolid residue (yield) was calculated and re-constituted in a measured amount of isotonic saline (NaCl 0.85%, *w/v*). The reconstituted extract concentration was adjusted at 15 and 30 mg/ml in isosaline; where a rat weighing 200 g receives 2 ml of the corresponding extract to be equivalent for the doses 150 and 300 mg/kg, respectively.

Yield % was calculated as: (extracted residue weight/original seed weight) × 100.

### Chemicals

Gentamicin was obtained as the patent preparation Garamycin® (Manufactured by: Memphis Co. for Pharm. & Chem. Ind. [MEMCO], Egypt; Under the authority of: Schering–Plough Corporation/U.S.A.; # 050039) that is formulated as ampoules of 1 ml containing 40 mg gentamicin as sulfate. The standard antanemic-antoxidant drug was obtained as the patent preparation Fefoli® (Metlar® Formulations, Chandigarh, India), which is formulated as 100 mg of ferrous ascorbate + 1.5 mg of folic acid per ml in a 150 ml bottle.hemoglobin Erythropoitin ELISA kit was purchased from HexaBiogen®, Ariana, Tunisia, for MyBioSource, Inc., CA, U.S.A. Iron-TIBC colorimetric reagent set was purchased from Pointe Scientific® Inc., MI, U.S.A. via a local distributer. Rat ferritin ELISA kit was purchased from Genway Biotec®, Inc., CA, U.S.A., via a local distributer. All other chemicals were of analytical grade and available from local distributers.

### Study design

Thirty male albino rats, weighing 180–200 g, were assigned to a parallel design in the present study. After acclimatization in hygienic condition with free access to clean water and balanced diet, the rats were randomly separated into five groups (six per group) in separate cages and then subjected to different treatments as follows: the Control group (C) was injected with sterile water (the vehicle of gentamicin) and orally administered with isosaline (the vehicle of *Carica papaya* leaf extract [*CPLE]*); the Diseased group (D) was injected with gentamicin (100 mg/kg, subcutaneously in the scruff region, for 21 consecutive days – and co-administered – orally, with the vehicle of *CPLE*; the Standard group (S) was injected with gentamicin in the same manner as that of group-D – and co-administered – orally, with a standard antanemic-antoxidant combination that is ferrous ascorbate–folic acid mixture in saline (13.5 + 0.135 mg/kg, respectively, daily for 21 days, using stomach tube); and the Treated groups (treated with small and large doses of CPLE [T_SD_ and T_LD_]), injected with gentamicin in the same manner as that of group-D, and co-administered orally with *CPLE* in isosaline (150 and 300 mg/kg, respectively, daily for 21 days, using stomach tube). Blood samples were collected 2 h post last administration, on day 21, from the retro-orbital venous plexus under light ether anesthesia. Two types of blood samples were saved, the first was received into EDTA tube for blood examination; while the second was received into a plain sampling tube, left to coagulate, centrifuged, and serum was harvested for determination of EPO, iron, UIBC, and ferretin. All procedures were ethical to animals and performed with merciful and humane manner under light ether anesthesia and adhered to the principles published by International Council for Laboratory Animal Science (ICLAS).

### Biochemical analysis

#### Hemogram

An auto-hematology analyser (Mindray®, Model BC-2800 Vet, Shenzhen, China) was used to determine erythrocytic parameters, including red blood cell (RBC) count, packed cell volume (PCV), hemoglobin concentration (HGB), mean cell volume (MCV), mean cell hemoglobin (MCH), and mean cell hemoglobin concentration (MCHC); leukocytic parameters, including total white blood cell (WBC) count, lymphocyte, granulocyte, monocytes, and platelets (PLT) counts [10].

#### EPO assay

Serum EPO level was determined as described previously [11] using rat EPO ELISA kit purchased from HexaBiogen® (Ariana, Tunisia, for MyBioSource, Inc., CA, U.S.A.) according to the instructions of the manufacturer. The intensity of the developed color was measured at 450 nm using ELISA microplate reader.

#### Iron/UIBC/TIBC assay

 Serum iron was determined according to the ferrozine principle described by Stookey [12], using a commercial diagnostic kit. Briefly, transferrin-bound iron in the sample (500 µl) was released at an acid pH (2.5 ml of hydroxylamine hydrochloride 220 mM in acetate buffer, pH 4.5) and reduced from ferric to ferrous status. These ions react with ferrozine (50 µl, 16.7 mM) to form a violet colored complex which was measured spectrophotometrically at 560 nm wavelength.

 TIBC was determined by calculation as the algebraic sum of the ‘serum iron concentration’ plus the ‘UIBC’ [13]. So, UIBC was determined first by ferrozine reaction after adding a known concentration (500 µl of the standard 500 µg/dl) of ferrous ions to the serum sample (500 µl) at an alkaline pH (Tris 500 mM, pH 8.1). The ferrous ions bind with transferrin at the unsaturated iron-binding sites. The additional unbound ferrous ions were measured using the ferrozine reaction (adding 50 µl of the iron color reagent, ferrozine 16.7 mM, and measuring the violet color intensity at 560 nm). The difference between the concentration of ferrous ions added and the unbound serum ions measured, was the UIBC.

 Transferrin saturation % was calculated as: (Serum iron [µg/dl]/TIBC [µg/dl]) × 100 [14].

 Serum transferrin concentration was calculated from its correlation with TIBC where one molecule of Transferrin can bind two molecules of iron at two high-affinity binding sites, using the following equation according to [15] and [16]: TIBC (µmol/l) = 25.1 × Transferrin (g/l).

Where, (µg/dl) = 0.179 (µmol/l) according to SI conversion.

#### Ferritin assay

Ferritin is a water soluble, iron storage protein. It was determined by ELISA using a commercial diagnostic kit according to the instructions of the manufacturer. Briefly, Ferritin present in the serum samples reacts with the anti-Ferritin antibodies which have been adsorbed to the surface of polystyrene microtiter well-plate. After the removal of unbound proteins by washing, anti-Ferritin antibodies conjugated with horseradish peroxidase (HRP), were added. These enzyme-labeled antibodies form complexes with the previously bound Ferritin. Following another washing step, the enzyme bound to the immunosorbent was assayed by the addition of a chromogenic substrate, 3,3’,5,5’-tetramethylbenzidine (TMB). The quantity of the bound enzyme varies directly with the concentration of Ferritin in the sample tested; thus, the absorbance using a microplate reader, at 450 nm, is a measure of the concentration of Ferritin in the test sample.

### Data presentation and analysis

Data are expressed as mean ± standard error of the mean of six separate observations. Observations were compared using analysis of variance (ANOVA) followed by least significant difference (LSD) as *post hoc* test at *P*-level of 0.05. All procedure of statistics and graphing were done using the computer program GraphPad Prism® version 6 (GraphPad Inc., CA, U.S.A.).

## Results

The yield % of the shade-dried *Carica papaya* leaves when macerated in hydroethanol (70:30, v/v) and evaporated was high, equals 32.4%. The extract was gummy in consistency and of green color.

Data of the present study showed that the daily administration of *CPLE* significantly affected both the erythrocyte and leukocyte parameters compared with the D-group (*P*>0.05). The effect was toward the physiological direction and its rate was directly proportional to the dose. Moreover, it was almost as that of the standard folate/ascorbate anti-anemic mixture used in the study. *CPLE* at small and large doses, significantly restored RBCs count, HGB, and PCV values that were deteriorated by successive administration of the nephrotoxin and gentamicin (Table 1).

**Table 1:** Erythrogram after adminstration of gentamicin and the protective role of *Carica papaya* leaf extract

Parameters	Groups				
	C	D	S	T_SD_	T_LD_
**RBC (10^12^/l)**	7.87 ± 0.27	4.97 ± 0.29^1^	7.17 ± 0.35^2^	6.78 ± 0.21^2^	7.10 ± 0.27^2^
**PCV (%)**	46.73 ± 2.43	28.33 ± 3.75^1^	41.87 ± 2.74^2^	38.67 ± 2.61^2^	41.34 ± 3.20^2^
**HGB (g/dl)**	14.07 ± 1.59	7.73 ± 0.72^1^	13.02 ± 0.95^2^	11.61 ± 0.93^2^	12.83 ± 0.91^2^
**MCV (fl)**	59.81 ± 2.93	57.03 ± 2.31	58.73 ± 2.35	57.14 ± 2.03	58.13 ± 2.17
**MCH (pg)**	18.21 ± 1.48	15.61 ± 1.28	18.03 ± 1.27	17.23 ± 1.18	17.84 ± 1.27
**MCHC (g/dl)**	30.47 ± 2.11	27.11 ± 1.75	30.68 ± 1.87	30.05 ± 2.07	30.85 ± 1.86

C: Control (saline was orally adminstered for 21 consecutive days); D: Diseased (gentamicin was s.c injected at dosage of 100 mg/kg b wt., for 21 consecutive days); T_SD_ or T_LD_: Treated with small or large dose (*Carica papaya* leaf extract was orally administered at dosage of 150 or 300 mg/kg b wt., for 21 consecutive days); S: Standard (Ferrous ascorbate + Folate as a combination of 13.5 + 0.135 mg/kg b wt. was orally administered for 21 consecutive days); Data are presented as Mean ± SE (*n*=6); ^1^ and ^2^ mean significantly (*P*<0.05) different from Normal and Diseased, respectively.

Both tested doses of *CPLE* had significant normalizing effects on most leukocytic parameters including total and granulocytes counts in addition to marked increase in platelet count compared with the D-group (Table 2).

**Table 2:** Leukogram after adminstration of gentamicin and the protective role of *Carica papaya* leaf extract

Parameters	Groups				
	C	D	S	T_SD_	T_LD_
**WBC (10^9^/l)**	14.63 ± 1.63	25.43 ± 2.43^1^	16.96 ± 1.09^2^	18.13 ± 1.20^2^	17.20 ± 1.36^2^
**Lymph. (%)**	72.93 ± 3.41	69.33 ± 3.75	72.46 ± 3.07	71.06 ± 2.39	72.27 ± 3.58
**Mid-sized (%)**	2.43 ± 0.61	1.79 ± 0.27	2.24 ± 0.41	2.07 ± 0.30	2.72 ± 0.29
**Granulocytes (%)**	24.36 ± 0.88	29.18 ± 1.59^1^	25.16 ± 1.74^2^	26.30 ± 1.46^2^	25.17 ± 1.17^2^
**PLT (10^9^/l)**	562.7 ± 7.84	481.67 ± 10.2^1^	546.3 ± 25.8	623.2 ± 39.2^2^	720.7 ± 52.9^2^

C: Control (saline was orally adminstered for 21 consecutive days); D: Diseased (gentamicin was s.c injected at dosage of 100 mg/kg b wt., for 21 consecutive days); T_SD_ or T_LD_: Treated with small or large dose (*Carica papaya* leaf extract was orally administered at dosage of 150 or 300 mg/kg b wt., for 21 consecutive days); S: Standard (Ferrous ascorbate + Folate as a combination of 13.5 + 0.135 mg/kg b wt. was orally administered for 21 consecutive days); Data are presented as Mean ± SE (*n*=6); ^1^ and ^2^ mean significantly (*P*<0.05) different from Normal and Diseased, respectively.

**Figure 2:**
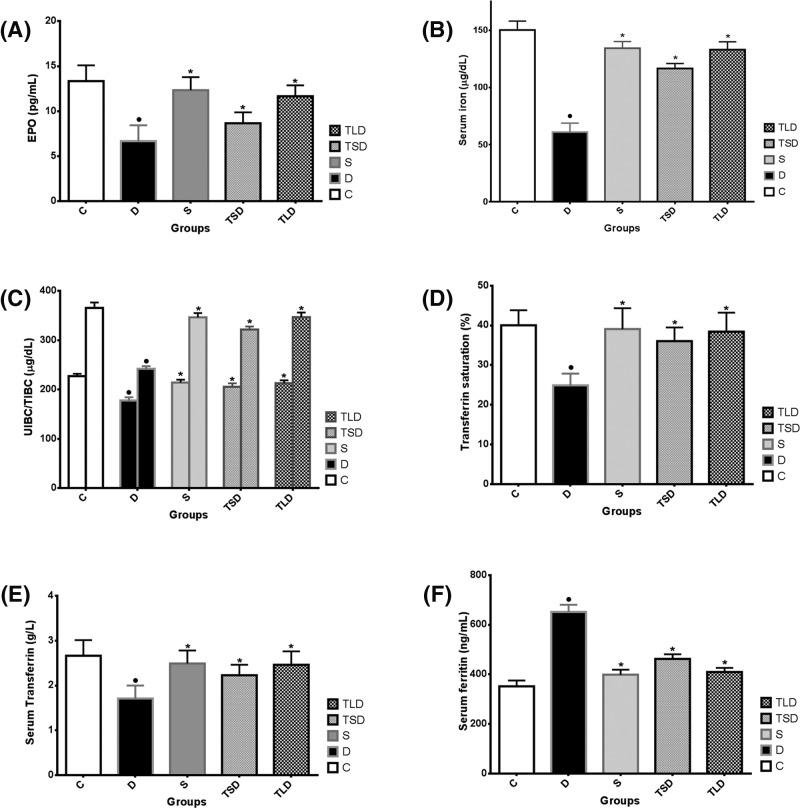
Erythropoietin, iron and iron indices after adminstration of gentamicin and the modulating role of *Carica papaya* leaf extract The recorded changes of EPO (**A**), Iron (**B**), UIBC/TIBC (**C**), Transferrin saturation % (**D**), and Transferrin (**E**) in rats after repeated subcutaneous injection of gentamicin (100 mg/kg b wt., for 21 consecutive days) and oral administration of *Carica papaya* leaf extract (150 and 300 mg/kg b wt., for 21 consecutive days) compared with those after the standard Ferrous ascorbate + Folate (13.5 + 0.135 mg/kg b wt., for 21 consecutive days) and normal control (Saline); (Mean ± SE; *n*=6). ^•^ and ^*^ mean significantly (*P*<0.05) different from Normal and Diseased, respectively. Abbreviations: C, control; D, diseased; S, standard.

Serum analysis assays revealed that *CPLE* significantly improved EPO concentration compared with that of D-group. Decreased serum iron, UIBC/TIBC, transferrin, and transferrin saturation % were significantly increased up to the normal levels by *CPLE*. On the other hand, the increased ferritin (a positive inflammatory reactant) concentration in D-group was almost corrected upon co-administration of *CPLE*, particularly at the larger dose level, with nephrotoxin (Figure 2A–F).

## Discussion

Iron deficiency anemia associated with inflammatory disorders, including kidney injury is attributed to rapid decline in renal function associated with rapid retention of nitrogenous waste products and decreased production of EPO, the main hormone of erythropoiesis [17]. Moreover, resistance to EPO or hypo-responsiveness to it in presence of inflammatory mediators associated with inflammatory disease conditions has been introduced to explain the inability to achieve and maintain the targeted blood parameter levels.

Gentamicin is one of the most common nephrotoxic models in rats [18]. Gentamicin binds to negatively charged phospholipids on tubular cell membranes and after internalization, it is transported to lysosomes causing reduced phospholipase activity and production of lipid peroxides [19].

Gentamicin-induced kidney disease has been utilized in the present study as a model for inflammatory renal anemia. Where, hematological and iron indices have been evaluated with or without co-administration of CPLE. Repeated gentamicin injections for 3 weeks resulted in significant reduction of RBC count, PCV, and HGB values in erythrogram, together with leuckocytosis, granulocytosis, and thrombocytopenia in leukogram, compared with those of the normal control; however, the rest of blood indices were minimally affected (Tables 1 and 2). The findings of erythrogram could be explained on the basis of the reduced EPO serum concentration determined in the present study (Figure 2A). Inflamed kidney may have a reduced capacity for production of adequate EPO. This finding is in agreement with [17] who reported that although the mechanisms involved in the pathogenesis of renal anemia are various, including inflammation, iron deficiency, and shortened half-life of erythrocytes, yet the primary cause is deficiency of EPO. In addition, inflammatory mediators and markers, such as C-reactive protein, hepcidin, and IL-6, were found to antagonize the response to EPO [20].

In addition to EPO, iron must be available for erythroid precursors in the bone marrow for erythropoiesis. In kidney disease, interference with iron metabolism from various aspects as well as inhibition of iron release from the reticuloendothelial system may occur. In the present study, serum iron level was found to be reduced in gentamicin group compared with the control group (Figure 2B). Hepcidin (a polypeptide hormone produced by the liver) plays a crucial rule in iron homeostasis. Inflammatory cytokines increase hepcidin production that decreases the efflux of recycled iron from splenic and hepatic macrophages, and the release of iron from storage in hepatocytes leading to functional iron deficiency contributing to renal anemia [5]. Hepcidin, in addition, binds to enterocytes causing poor absorption of dietary iron.

Transferrin is a protein synthesized and released by the liver, responsible for iron transport through the plasma. Usually, it increases in absolute iron deficiency to maximize utilization of the little available iron [21]. However, in inflammation, the diseased liver ability to synthesize proteins, including transferrin, is reduced. In the same line, UIBC and TIBC could be alternative tests to transferrin. UIBC/TIBC and transferrin saturation % give indication about the free iron binding sites on transferrin. Again, usually these values increase in absolute iron deficiency but decrease in iron overload [22]. However, in the present study all values were decreased despite the recorded reduction of serum iron because of the reduced transferrin concentration itself (Figure 2C–E) under the induced inflammatory condition. Therefore, iron and iron indices are considered as good tools for accurate etiologic diagnosis of anemias associated with iron deficiency [23]. Additional valuable test that differentiates between anemic conditions characterized by iron deficiency (whether absolute iron deficiency anemia or inflammatory anemia), is ferritin. Ferritin is the second major iron-containing protein, after hemoglobin, in the body. However, unlike hemoglobin, it acts only as iron store found mainly in the cytoplasm of reticuloendothelial cells [24], and in the liver, spleen, bone marrow, and serum. Direct correlation exists between serum ferritin and total iron storage in the body. It is assumed that for each 1 ng/ml of serum ferritin, 10 mg of iron is stored in tissues and organs [25]. Decreased serum iron together with depleted ferritin under physiological levels is indicative of absolute iron deficiency anemia. However, decreased serum iron level together with high ferritin in the context of a decreased transferrin saturation is indicative for immune-driven iron sequestration as occuring in inflammation, infection, liver or kidney disease, and cancer [26]. This statement is in complete accordance with iron and ferritin findings of the present study, where serum iron was found to be lower, while ferritin was found to be higher, both along with lower UIBC/TIBC and transferrin saturation % (Figure 2) confirming the type of anemia recorded in the present study is inflammatory anemia rather than iron deficiency anemia.

The antanemic and hematoprotective results of *CPLE* recorded in the present study may be explained by its nephroprotective potential driven from its antioxidant properties of contained phytochemicals [8]. By the occurred renal protection, the critical hematopoietic role of the kidney was preserved-indicated by improved blood picture (Tables 1 and 2). The extract was found to significantly restore the reduced values of RBCs, PCV, and hemoglobin up to the normal levels. The effects of the extract were comparable to those of the standard anti-anemic mixture used. These findings are in agreement with [27], who found that ethanolic CPLE increased blood parameters, namely, RBC count, PCV, and HGB in rats especially at higher doses. However, the authors found that the seed and pulp extracts reduced these parameters with no significant effects at smaller doses. Our findings are also consistent with those of [28] who reported that 5% dried pawpaw seed meal tended to improve hematological parameters in broilers fed on it. On the other hand, the present results may disagree with those of [29] who reported that *Carica papaya* bark affects adversely on blood and biochemical parameters of treated rats; and with those of [30] who found that *Carica papaya* leaf meal has no strong hematological attribute as feedstuff in the diet of *Heterobranchus bidorsalis–Clarias gariepinus* hybrid. Such discrepancy between our findings and those of other authors might be attributed to the different parts used of the plant, the different experimental animals, the different methodology adopted as well as dosage followed.

As for leukogram, data of the present study proved that CPLE significantly increased total WBC count with granulocytosis and thrombocytopenia. These findings of WBCs count are in accordance with those of [29] and [31] in rats and humans, respectively. The marked increase in platelet count recorded in the present study was evident in previous studies in human [31] and murine model [32] as well. It is suggested that there could be some active compounds in CPLE that can enhance thrombopoiesis. Chemical analysis of CPLE showed the presence of considerable amounts of carpaine, malic acid, quinic acid, manghaslin and clitorin, minor quantities of various malic acid derivatives, nicotiflorin, rutin, and unidentified constituents. The leaf extract is; therefore, highly recommended for treatment of Dengue fever that is characterized by thrombocytopenia [33] and [34].

Data of the present study proved that the protective effect of CPLE on the kidney tissue and guarding against the advancement of the disease was reflected greatly on the blood picture of the treated animals. Although there are no previous studies on the effects of CPLE on EPO and iron indices, yet our findings regarding these valuable parameters may be explained by the virtue of kidney tissue protection, especially the tubular part. *CPLE* reserved the hemopoietic function of the kidney and maintained the targeted EPO synthesis and release. From another aspect of view, the anti-inflammatory potential of the plant leaf extract added more to the reduction of the inflammatory response induced by gentamicin. Interference with the inflammatory mediators reduced their negative effect on EPO from one hand and normalized ferritin (a positive inflammatory reactant) and iron indices from another hand. The anti-inflammatory and immunomodulatory effects of *Carica papaya* were reviewed by [35], both *in vitro* and *in vivo*.

## Conclusion

From the above mentioned findings and explanations, it could be concluded that the aqueous extract of *Carica papaya* leaves has a strong potential of hemoprotection – driven from its nephroprotective effect – and its content of beneficial phytochemical constituents. The leaves of *Carica* papaya plant, thus, could be a good pharmaceutical source of natural hematopoietic medicines in conditions associated with inflammatory anemias particularly renal ones.

### Clinical significance

Findings of the present study provide two clinical impacts, diagnostic and therapeutic. It demonstrates the inflamed kidney-driven renal anemia in terms of hematological parameters and iron indices, including serum iron, ferritin, transferrin, UIBC/TIBC, and transferrin saturation %; and give evidence for *Carica papaya* leaf extract as a natural source for a promising antanemic pharmaceutical preparation in context of nephroprotection and scavenging of oxidative stress mediators.
